# Statin use and mortality risk in Asian patients with prostate cancer receiving androgen deprivation therapy: A population‐based cohort study

**DOI:** 10.1002/cam4.6826

**Published:** 2023-12-22

**Authors:** Yan Hiu Athena Lee, Jeffrey Shi Kai Chan, Jeremy Man Ho Hui, Pias Tang, Kang Liu, Edward Christopher Dee, Kenrick Ng, Gary Tse, Chi Fai Ng

**Affiliations:** ^1^ Division of Urology, Department of Surgery, Faculty of Medicine The Chinese University of Hong Kong Hong Kong China; ^2^ Cardio‐Oncology Research Unit, Cardiovascular Analytics Group PowerHealth Limited Hong Kong China; ^3^ Quest Genomics Limited London United Kingdom; ^4^ Department of Radiation Oncology Memorial Sloan Kettering Cancer Center New York New York USA; ^5^ Department of Medical Oncology University College London Hospitals NHS Foundation Trust London United Kingdom; ^6^ Department of Cardiology, Tianjin Key Laboratory of Ionic‐Molecular Function of Cardiovascular Disease Tianjin Institute of Cardiology, Second Hospital of Tianjin Medical University Tianjin China; ^7^ Kent and Medway Medical School Canterbury United Kingdom; ^8^ School of Nursing and Health Studies Hong Kong Metropolitan University Hong Kong China; ^9^ SH Ho Urology Centre The Chinese University of Hong Kong Hong Kong China

**Keywords:** clinical cancer research, clinical observations, prostate cancer

## Abstract

**Background:**

This study aimed to examine the associations between the use of statins concurrent with androgen deprivation therapy (ADT) and the risks of mortality in Asian patients diagnosed with prostate cancer (PCa).

**Methods:**

Adult patients (≥18 years old) diagnosed with PCa who were receiving any form of ADT and were being treated at public hospitals in Hong Kong from December 1999 to March 2021 were retrospectively identified, with follow‐up conducted until September 2021. Patients who had received medical castration for <180 days without subsequent bilateral orchidectomy, those who had used statins concurrently with ADT for <180 days, and those with missing baseline total cholesterol levels were excluded. Statin users were defined as individuals who had used statins for ≥180 days concurrent with ADT, while non‐users were those who had not used any statins. PCa‐related mortality was the primary outcome, while all‐cause mortality served as the secondary outcome. Inverse probability treatment weighting was employed to balance the covariates.

**Results:**

A total of 4920 patients were included, consisting of 2578 statin users and 2342 non‐users (mean age 76.1 ± 8.2 years). Over a mean follow‐up period of 4.2 ± 3.3 years, it was observed that statin users had significantly lower risks of both PCa‐related mortality (weighted hazard ratio [wHR] 0.56 [95% confidence interval (CI) 0.48, 0.65], *p* < 0.001) and all‐cause mortality (wHR 0.57 [95% CI 0.51, 0.63], *p* < 0.001), regardless of the type of ADT used. Notably, these associations were more pronounced among patients with less advanced PCa, as indicated by the absence of androgen receptor antagonist or chemotherapy usage (*p* value for interaction <0.001 for both outcomes).

**Conclusion(s):**

The use of statins concurrent with ADT was associated with reduced mortality risks among Asian patients with PCa. These findings suggest the need for additional research to explore the potential role of statins in the treatment of PCa patients.

## Graphical abstract

The use of statins was associated with reduced risks of prostate cancer‐related mortality and all‐cause mortality among patients receiving androgen deprivation therapy for prostate cancer. These associations were more pronounced in patients who did not use androgen receptor antagonists or undergo chemotherapy.

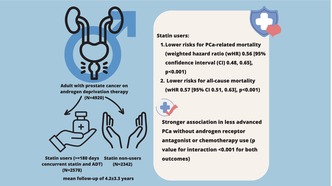

## 1 INTRODUCTION

In 2012, prostate cancer (PCa) ranked as the second most prevalent cancer and the fifth leading cause of death globally among males.^1^ Moreover, there has been a noticeable rise in the incidence of PCa in Asian countries in recent times.^2^ Over the past few years, several studies have demonstrated the potential anti‐cancer effects of statins, and while their use as cancer therapy is only hypothesis‐generating at this time, ongoing research is essential.^3^, ^4^ Retrospective analyses suggested that statins confer survival benefit in gastric cancers and triple‐negative breast cancers.^5^, ^6^ At present, the STAT‐ROC phase III trial (ISRCTN98060456) is actively enrolling participants with the objective of evaluating the impact of adjuvant statins in patients who have undergone curative surgery for esophageal adenocarcinomas.^7^

However, evidence underlying the associations between statin use during androgen deprivation therapy (ADT), which is the primary treatment for metastatic PCa with or without definitive radiotherapy,^8^, ^9^ and the associated risks of mortality in PCa patients remains uncertain. Although preclinical studies have indicated that statins can induce apoptosis in tumor cells specific to PCa and enhance their invasiveness,^10^, ^11^ a recent meta‐analysis revealed that the use of statins did not show a significant association with PCa‐specific survival in patients undergoing ADT, despite an improvement in overall survival.^12^ Nonetheless, significant inter‐study differences prevented conclusions from being drawn,^4^ and studies are needed to identify if statins can improve mortality risk in PCa patients. Therefore, this study aimed to examine the associations between the concurrent use of statins and ADT and the risks of PCa‐related mortality and all‐cause mortality in Asian patients diagnosed with PCa.

## 2 METHODS

This retrospective cohort study was conducted in accordance to the Declaration of Helsinki and followed the STROBE guideline.^13^ Approval for the study was obtained from the Joint Chinese University of Hong Kong—New Territories East Cluster Clinical Research Ethics Committee. Patient consent was not required because of the use of deidentified data. Access to the data supporting this study is available upon reasonable request to the corresponding author.

### 2.1 Source of data

All the data used in this study were obtained from the Clinical Data Analysis and Reporting System (CDARS), which is an electronic health records database linked to the Hospital Authority of Hong Kong. This comprehensive system captures essential information such as demographics, diagnoses, procedures, and medication records of patients receiving care in public healthcare institutions across Hong Kong. The *International Classification of Diseases, Ninth Revision* (ICD‐9) codes are employed to code all diagnoses. Medication records, including drug name, drug item code, dispensing date, route and dose of each prescription, and prescription interval, are automatically recorded by CDARS. Furthermore, CDARS is connected to the Hong Kong Death Registry, which is a comprehensive population‐wide governmental registry. The causes of mortality are recorded using either ICD‐9 or ICD‐10, depending on the year of death. CDARS, along with its linked mortality data, has been widely used for research purposes.^14^, ^15^, ^16^, ^17^ Our team has previously used this cohort of PCa patients receiving ADT for studying cardiovascular outcomes.^18^, ^19^, ^20^

### 2.2 Patient population

This study consisted of adult patients (≥18 years old) who had been diagnosed with PCa and were receiving any form of ADT in Hong Kong between December 1999 and March 2021. The diagnosis of PCa was confirmed using ICD‐9 codes (Table S1). ADT encompassed bilateral orchidectomy, gonadotrophin‐releasing hormone agonists, and gonadotrophin‐releasing hormone antagonists. Exclusions were made for the following cases: (a) patients who had received <180 days of medical castration without subsequent bilateral orchidectomy; (b) patients who had <180 days of concurrent statin use and ADT; and (c) patients with missing baseline total cholesterol levels.

### 2.3 Definition of statin users and non‐users

Patients classified as statin users were those who had used statins concurrently with ADT for ≥180 days. On the other hand, statin non‐users were defined as patients who had never used statins.

### 2.4 Follow‐up and outcomes

All included patients were followed up from the date of initiating ADT, which served as the baseline date, until September 30, 2021. The primary outcome of the study was PCa‐related mortality, while all‐cause mortality served as the secondary outcome. The time period between ADT initiation and mortality was documented, and the causes of death were determined using ICD codes (Table S2).

### 2.5 Covariates

Baseline information for all included patients, encompassing their age, the specific type of ADT received, and the presence of various comorbidities determined by ICD‐9 codes, was recorded. These comorbidities included hypertension, ischaemic heart disease, myocardial infarction, heart failure, stroke, diabetes mellitus, chronic kidney disease, anemia, atrial fibrillation, chronic liver disease, chronic obstructive pulmonary disease, hyperlipidaemia, and any malignancy (specific codes can be found in Table S1). The use of other medications was also documented, such as angiotensin‐converting enzyme inhibitors or angiotensin II receptor blockers, beta‐blockers, dihydropyridine calcium channel blockers, metformin, sulfonylureas, dipeptidyl peptidase‐4 inhibitors, glucagon‐like peptide 1 receptor agonists, insulin, corticosteroids, antiplatelets, anticoagulants, and androgen receptor antagonists (abiraterone, enzalutamide, and bicalutamide). Furthermore, the patients' history of undergoing radiotherapy, radical prostatectomy, prior chemotherapy (including docetaxel, cabazitaxel, mitoxantrone, and estramustine), chemotherapy concurrent with ADT, and their baseline total cholesterol level were recorded. Additionally, the average daily dose of statin used was documented and converted to a simvastatin‐equivalent dose using the anatomical therapeutic chemical classification and the defined daily dose recommended by the World Health Organization Collaborating Centre for Drug Statistics Methodology.

### 2.6 Analysis or statistical methods

Continuous variables were presented as mean ± standard deviation. To achieve balance between the treatment groups, logistic regression‐based inverse probability treatment weighting (IPTW) was employed using the aforementioned covariates. The standardized mean difference (SMD) was used to assess the balance of covariates between the treatment groups, with values <0.1 indicating favorable balance.

IPTW‐weighted univariable Cox regression was used to evaluate the association between the use of statins and the risks of the outcomes. As summary statistics, we calculated weighted hazard ratios (wHR) along with 95% confidence intervals (CI). Kaplan–Meier curves were generated to visualize the cumulative freedom from the outcomes.

All *p*‐values were two‐sided, and statistical significance was considered for values below 0.05. The statistical analyses were conducted using SPSS (version 25.0, IBM Corp., USA) or Stata (version 13.0, StataCorp LLC, USA).

### 2.7 Subgroup analyses

Because of the limitations inherent in our data source, information regarding the staging and grading of cancer is not accessible. To address this limitation, we considered the use of androgen receptor antagonists or chemotherapy, which are common treatments for metastatic PCa, as a surrogate marker for metastatic PCa.^21^ An a priori subgroup analysis was performed to compare patients who received these medications with those who did not, aiming to investigate whether the associations between statin use and mortality risks would apply specifically to patients with metastatic PCa. Additionally, a separate a priori subgroup analysis was conducted for each type of ADT administered to assess whether the associations between statin use and mortality risks remained significant across different types of ADT.

To explore the association between statin dosage and the risk of mortality, an additional a priori subgroup analysis was performed. This analysis involved comparing patients who were categorized as either receiving a high dosage or a low dosage of statins with those who did not use statins at all. Statin users were divided into high‐dose and low‐dose groups based on the median dosage.

### 2.8 Sensitivity analyses

To examine the impact of statin use during the initiation of ADT on the observed outcomes, a sensitivity analysis was performed. In this analysis, patients who were not using statins at the time of ADT initiation were excluded from the group of statin users. As a result, a comparison was made between statin users who had initiated statin use at the time of ADT initiation and continued using it for ≥6 months alongside ADT and patients who had never used statins.

Considering the potential synergistic impact of ADT and radiation therapy, a sensitivity analysis was performed including only patients who did not receive radiation therapy. Additionally, a sensitivity analysis was conducted including only patients who did not receive radical prostatectomy. Furthermore, another sensitivity analysis was performed including only patients who did not either receive radiotherapy, radical prostatectomy, or a combination of both treatments. The aim of these analyses was to determine if the associations between statin use and mortality risks would be applicable to patients in these different treatment groups.

Although the current study encompassed both hydrophilic and lipophilic statins, a sensitivity analysis was conducted to focus specifically on the mortality effects of lipophilic statin use. This analysis involved excluding patients who had any exposure to hydrophilic statins from the statin user group. The aim was to examine the impact of lipophilic statin use on mortality outcomes.

Furthermore, a sensitivity analysis was performed including only patients who had a follow‐up period of ≥3 years.

As a post hoc sensitivity analysis, an unweighted multivariable Cox regression was performed. Hazard ratios (HR) along with 95% CI were used as summary statistics.

To address the potential influence of baseline PSA on the observed associations, an additional sensitivity analysis was conducted. This post hoc analysis involved adjusting for baseline PSA, when available, using multivariable Cox regression.

Because of the high mortality rate within the cohort, we aimed to mitigate the potential inaccuracies in hazard estimation that may arise from conventional survival analyses. To achieve this, we employed a competing risk analysis methodology. Specifically, a fine‐gray sub‐distribution model was used, considering non‐PCa‐related mortality as the competing event. Univariable competing risk regression with IPTW was used to evaluate the association between statin use and the risk of PCa‐related mortality. As summary statistics, we calculated sub‐hazard ratios (SHR) with 95% CI.

## 3 RESULTS

**FIGURE 1:**
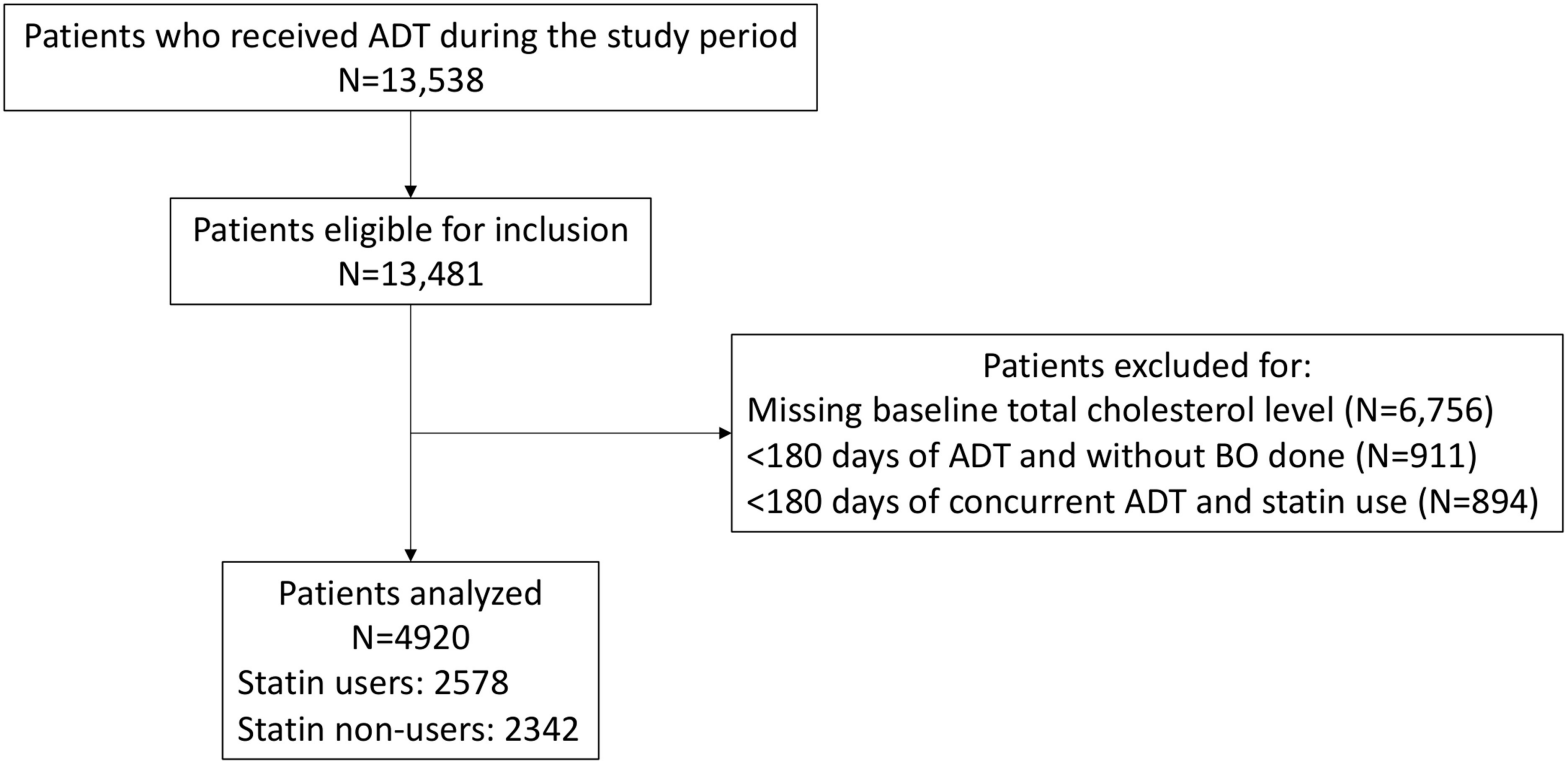
Study flow chart. ADT, androgen deprivation therapy; BO, bilateral orchidectomy.

A total of 13,481 patients met the inclusion criteria initially. However, after applying the exclusion criteria, the study cohort comprised of 4920 patients (Figure 1). Among these patients, 2578 were classified as statin users, while 2342 were categorized as non‐users. The average simvastatin‐equivalent daily dose of statin used was 18.9 ± 2.0 mg/day. The mean age was 76.1 ± 8.2 years. Out of the total patients, 2870 (58.3%) only received medical castration, 1681 (34.2%) only received bilateral orchidectomy as ADT, and 369 (7.5%) received both treatments. Among those who underwent medical castration alone, the average treatment duration was 3.1 ± 2.5 years. Table 1 summarizes the baseline characteristics of all included patients, demonstrating well‐balanced covariates achieved through IPTW (SMD < 0.1 for all variables).

**TABLE 1:** Baseline characteristics with standardized mean differences (SMD) before and after applying inverse probability treatment weighting (IPTW).

	Statin non‐users (*N* = 2342)	Statin users (*N* = 2578)	Unweighted SMD	SMD with IPTW
Age, years	76.4 ± 8.6	75.8 ± 7.8	0.08	<0.01
Use of GnRH agonist or antagonist, *N* (%)	1418 (60.5)	1821 (70.6)	0.21	<0.01
Bilateral orchidectomy, *N* (%)	1195 (51.0)	855 (33.2)	0.37	<0.01
Hypertension, *N* (%)	759 (32.4)	1263 (49.0)	0.34	<0.01
Ischaemic heart disease, *N* (%)	148 (6.3)	693 (26.9)	0.57	0.07
Myocardial infarction, *N* (%)	46 (2.0)	232 (9.0)	0.31	0.07
Heart failure, *N* (%)	147 (6.3)	207 (8.0)	0.07	0.04
Stroke, *N* (%)	189 (8.1)	471 (18.3)	0.30	<0.01
Diabetes mellitus, *N* (%)	539 (23.0)	1154 (44.8)	0.47	0.03
Chronic kidney disease, *N* (%)	90 (3.8)	140 (5.4)	0.08	0.04
Anemia, *N* (%)	215 (9.2)	202 (7.8)	0.05	0.03
Atrial fibrillation, *N* (%)	144 (6.1)	190 (7.4)	0.05	0.05
Chronic liver disease, *N* (%)	35 (1.5)	40 (1.6)	<0.01	0.01
Chronic obstructive pulmonary disease, *N* (%)	156 (6.7)	129 (5.0)	0.07	0.01
Hyperlipidaemia, *N* (%)	91 (3.9)	721 (28.0)	0.69	<0.01
Ever underwent radiotherapy, *N* (%)	490 (20.9)	477 (18.5)	0.06	<0.01
Ever underwent radical prostatectomy, *N* (%)	772 (33.0)	753 (29.2)	0.08	<0.01
Any malignancy, *N* (%)	344 (14.7)	249 (9.7)	0.16	<0.01
ACEI/ARB use, *N* (%)	693 (29.6)	1371 (53.2)	0.49	<0.01
Beta‐blocker use, *N* (%)	864 (36.9)	1427 (55.4)	0.38	0.03
Dihydropyridine calcium channel blocker use, *N* (%)	1232 (52.6)	1673 (64.9)	0.25	0.02
Metformin use, *N* (%)	278 (11.9)	720 (27.9)	0.41	0.03
Sulfonylurea use, *N* (%)	331 (14.1)	689 (26.7)	0.31	0.04
DPP‐4 inhibitor use, *N* (%)	20 (0.9)	90 (3.5)	0.18	0.06
GLP‐1 receptor agonist use, *N* (%)	0 (0)	2 (0.1)	0.04	0.03
Insulin use, *N* (%)	153 (6.5)	279 (10.8)	0.15	0.04
Corticosteroid use, *N* (%)	452 (19.3)	476 (18.5)	0.02	0.02
Antiplatelet use, *N* (%)	491 (21.0)	1265 (49.1)	0.61	0.06
Anticoagulant use, *N* (%)	79 (3.4)	157 (6.1)	0.13	<0.01
Androgen receptor antagonist use, *N* (%)	940 (40.1)	1235 (47.9)	0.16	0.01
Prior chemotherapy, *N* (%)	11 (0.5)	15 (0.6)	0.02	0.02
Chemotherapy concurrent with ADT, *N* (%)	238 (10.2)	265 (10.3)	<0.01	<0.01
Total cholesterol, mmol/L	4.6 ± 0.9	4.2 ± 1.0	0.35	0.06

Abbreviations: ACEI, angiotensin converting enzyme inhibitor; ADT, androgen deprivation therapy; ARB, angiotensin receptor blocker; GnRH, gonadotropin hormone‐releasing hormone; HbA1c, hemoglobin A1c.

**FIGURE 2:**
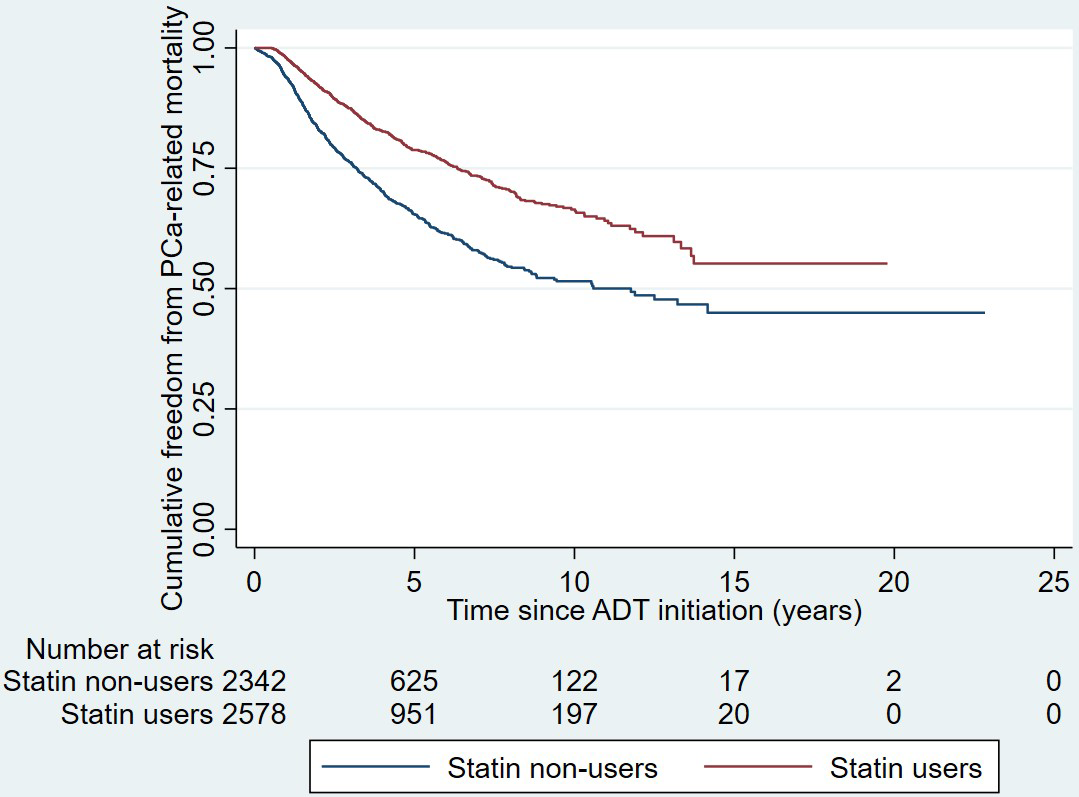
Kaplan–Meier curve illustrating the cumulative freedom from PCa‐related mortality. ADT, androgen deprivation therapy; PCa, prostate cancer.

**FIGURE 3:**
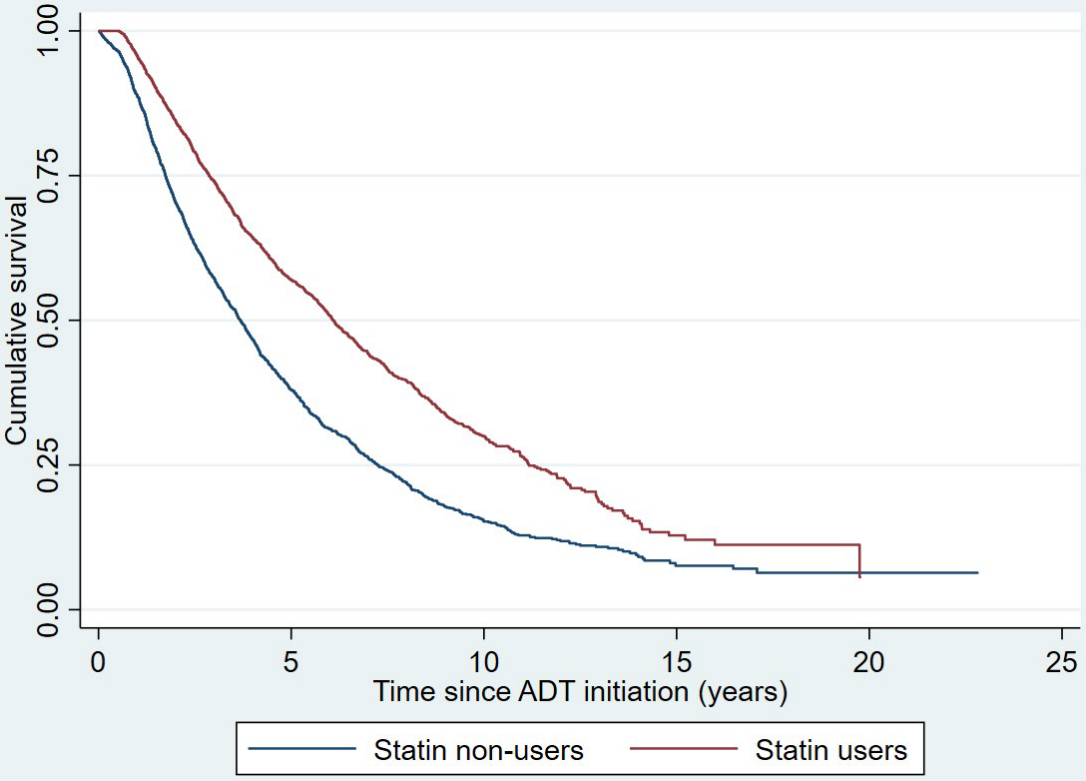
Kaplan–Meier curve illustrating the cumulative freedom from all‐cause mortality. ADT, androgen deprivation therapy.

During an average follow‐up period of 4.2 ± 3.3 years, 1206 patients (14.5%) experienced PCa‐related mortality, while 2944 patients (59.8%) experienced all‐cause mortality. Overall, the use of statins was associated with significantly reduced risks of both PCa‐related mortality (wHR 0.56 [0.48, 0.65], *p* < 0.001; as shown in Figure 2) and all‐cause mortality (wHR 0.57 [0.51, 0.63], *p* < 0.001; as shown in Figure 3).

### 3.1 Subgroup analyses

Statin users had significantly reduced risks of PCa‐related mortality as well as all‐cause mortality, irrespective of whether they received androgen receptor antagonists or chemotherapy (*N* = 1872) or not (*N* = 2620). However, the beneficial effects of statins were more pronounced in patients without androgen receptor antagonist or chemotherapy use, as evidenced by a *p*‐value for interaction of less than 0.001 for both outcomes (as shown in Table 2). This finding suggests that the survival advantages associated with statins may be particularly notable among patients without metastatic PCa.

**TABLE 2:** Weighted comparisons of outcomes by statin use with subgroups for androgen receptor antagonist or chemotherapy use.

	No androgen receptor antagonist or chemotherapy use (*N* = 2620)		Androgen receptor antagonist or chemotherapy use (*N* = 1872)		*p* Value for interaction
	wHR [95% CI]	*p* Value	wHR [95% CI]	*p* Value	
Prostate cancer‐related mortality	0.39 [0.31, 0.50]	<0.001	0.69 [0.56, 0.87]	0.001	<0.001
All‐cause mortality	0.47 [0.41, 0.55]	<0.001	0.69 [0.60, 0.80]	<0.001	<0.001

*Note*: Weighted hazard ratios (wHR) with corresponding 95% confidence intervals (CI) were calculated, using statin non‐users as the reference group.

Statin users experienced significantly reduced risks of PCa‐related mortality and all‐cause mortality, regardless of whether they underwent bilateral orchidectomy only (*N* = 1681) or medical castration only (*N* = 2870), as shown in Table S3. Although there were numerical trends indicating lower mortality risks, the statistical significance for patients who received both treatments may have been diminished due to the small number of patients in this particular subgroup (*N* = 369).

Compared to individuals who did not use statins, the use of statins at any dose was associated with significantly reduced risks of PCa‐related mortality (low‐dose: HR 0.56 [0.48–0.65], *p* < 0.001; high‐dose: HR 0.51, 95% CI [0.43–0.61], *p* < 0.001) and all‐cause mortality (low‐dose: HR 0.55, CI [0.49–0.60], *p* < 0.001; high‐dose: HR 0.56, 95% CI [0.50–0.62], *p* < 0.001). The 95% CIs for these associations were overlapping.

### 3.2 Sensitivity analyses

The association between statin use and decreased risks of both outcomes remained significant when comparing statin users who had initiated statin use at the time of ADT initiation with patients who had never used statins (*N* = 4436; *p* < 0.001 for both outcomes), as shown in Table S4.

Consistent findings were observed in statin users who did not receive radiotherapy, where statin use was linked to reduced risks of PCa‐related mortality (HR 0.61 [0.53, 0.69], *p* < 0.001) as well as all‐cause mortality (HR 0.63 [0.58, 0.69], *p* < 0.001). Similarly, statin users who did not receive radical prostatectomy experienced significantly lower risks of PCa‐related mortality (HR 0.51 [0.44, 0.59], *p* < 0.001) and all‐cause mortality (HR 0.58 [0.53, 0.63], *p* < 0.001). These associations persisted in patients who did not either receive radiotherapy, radical prostatectomy, or a combination of both treatments, where statin use was linked to decreased risks of both PCa‐related mortality (HR 0.55 [0.48, 0.62], *p* < 0.001) and all‐cause mortality (HR 0.60 [0.55, 0.64], *p* < 0.001). These findings suggest that the associations between statin use and mortality risks are applicable to patients in these different treatment groups.

Furthermore, the association between statin use and reduced risks of mortality outcomes remained significant when the analysis specifically focused on the mortality effects of lipophilic statin use (*N* = 4686; *p* < 0.001 for both outcomes), as shown in Table S5.

After adjusting the time interval to include only patients with a follow‐up period of ≥3 years (*N* = 2661), statin use remained associated with significantly reduced risks of PCa‐related mortality (HR 0.68 [0.52, 0.88], *p* = 0.003) and all‐cause mortality (HR 0.67 [0.58, 0.76], *p* < 0.001), as summarized in Table S6.

Consistent findings were observed when multivariable Cox regression was employed instead of using IPTW. In this analysis, statin use remained associated with reduced risks of PCa‐related mortality (HR 0.54 [0.47, 0.61], *p* < 0.001) and all‐cause mortality (HR 0.55 [0.51, 0.60], *p* < 0.001).

In the subset of 4818 patients who had baseline PSA levels available, the association between statin use and reduced risks of PCa‐related mortality (HR 0.56 [0.49, 0.64], *p* < 0.001) and all‐cause mortality (HR 0.56 [0.51, 0.61], *p* < 0.001) persisted even after adjusting for baseline PSA levels using multivariable Cox regression.

Moreover, our findings were supported by robust results from univariable competing‐risk regression analysis. Statin use continued to be associated with reduced risks of PCa‐related mortality (SHR 0.66 [0.57, 0.77], *p* < 0.001) with non‐PCa‐related mortality considered as the competing event.

## 4 DISCUSSION

In this population‐based retrospective cohort study, it was found that the concurrent use of statins and ADT in an Asian population with PCa was associated with significantly reduced risks of both PCa‐related mortality and all‐cause mortality. The associations were more pronounced in patients who did not receive androgen receptor antagonist or chemotherapy treatment.

### 4.1 Underlying mechanisms

The primary mechanism by which statins inhibit the growth of PCa is through the reduction of androgen receptor (AR) signaling independently of the levels of circulating androgens, which is mediated by cholesterol.^22^ PCa cells proliferate in an androgen‐sensitive manner, and the activation of AR alters cell cycle control^23^ and increases oncogene expression by direct interaction with transcriptional cofactors.^24^ AR signaling is vital in PCa progression regardless of castration status,^25^ which underlies ADT as the first‐line treatment of PCa. Statins could be a useful adjuvant to ADT by further dampening AR signaling. Moreover, statins have cholesterol‐lowering properties achieved by inhibiting 3‐hydroxy‐3‐methylglutaryl–coenzyme A reductase. This action disrupts the organization of lipid rafts,^26^ specialized domains in the cell membrane enriched with cholesterol that facilitate the signaling pathways of membrane receptors like AR.^27^ As a result, statins may impede the survival and proliferation of PCa cells. Additionally, statins counteract the increased activity of intracellular cholesterol metabolism observed in castration‐resistant prostate cancer (CRPC) cell lines, which plays a crucial role in the development of resistance to ADT during prolonged usage.^28^ Therefore, the cholesterol‐lowering ability of statins is desirable in delaying the development of CRPC, a condition with limited treatment options and higher risks of mortality.^29^

The beneficial effects of statins in patients with PCa may not solely rely on cholesterol‐mediated mechanisms. Other pathways or mechanisms independent of cholesterol could also play a role in the protective effects of statins. In vitro studies showed that statins competitively reduce dehydroepiandrosterone sulfate (DHEAS) uptake. Since DHEAS is a substrate for testosterone synthesis, statins can effectively reduce the level of intratumoral androgen.^30^ In addition, statins are well‐known for their apoptosis‐inducing effects on tumor cells. With less mevalonate activating cyclin‐dependent kinase 2, cell cycle progression in PCa cells is reduced.^10^ Lower levels of mevalonate also lead to reduced inhibition and thus, increased activity of caspace‐7, a critical protease in apoptotic pathways.^31^ The apoptosis‐inducing ability of statins is further attributable to a downregulation of phosphorylation pathways mediated by AKT kinase in PCa cells.^11^ These non‐cholesterol‐mediated mechanisms may thereby confer antineoplastic properties in PCa through their intramural androgen‐lowering and apoptotic effects.

### 4.2 Prior studies and future directions

Although certain studies have proposed a potential association between the use of statins and enhanced survival outcomes in patients undergoing ADT, contrasting findings have also been reported.^32^, ^33^ Mikkelsen et al.^32^ conducted a study that found no association between statin use at the time of PCa diagnosis and the time to progression, which was defined as the development of CRPC or PCa‐related death, among patients receiving ADT. It is important to note that this study had limitations due to its small sample size of 537 participants. While Mikkelsen et al. suggested that selection bias may have influenced the observed protective effect of statins against mortality in previous studies, as statin users were typically more health‐conscious and had a lower incidence of metastasis due to access to improved treatment options, referred to as the “healthy user effect”,^32^ a different large observational study involving a significant sample size of 87,346 individuals reported contrasting findings. In this study, it was observed that statin users were actually more prone to having a higher Charlson Comorbidity Index and being diagnosed with high‐grade cancer.^34^ Ultimately, the risk of a “healthy user effect” can only be thoroughly eliminated by randomized controlled trials (RCTs). A small RCT by Murtola et al.^35^ attempted to address the role of statins in patients with PCa, with initial findings suggesting that atorvastatin does not lower the proliferation rate of PCa. Nonetheless, this trial did not investigate effect of statins on mortality, and the short duration of statin exposure (median of 27 days) severely limited its clinical implications. Further clinical trials are necessary to gain a clearer understanding of the relationship between statin usage and mortality risks in individuals with PCa. The ongoing PEACE‐4 trial (registered as NCT03819101) is a phase III RCT employing a 2 × 2 factorial design. Its primary objective is to assess the impact of acetylsalicylic acid and atorvastatin on overall survival in patients with CRPC who are initiating first‐line treatment. The results of this trial are expected to offer valuable insights into the aforementioned topic.

In addition, few studies have investigated the effect of statin usage at different times relative to ADT. Peltomaa et al.^33^ discovered that the use of statins following the initiation of ADT, but not prior to it, was linked to a reduced risk of PCa mortality. However, it is important to approach these findings cautiously because the study could not account for the various factors that may have contributed to discontinuation of statins, such as having poor lipid‐wise treatment effects, adverse reactions to statins, or requiring other medications that may interact with statins, all of which may confound the above observations. This issue was circumvented in the present study by only including patients with statin use concurrent with ADT. Furthermore, the sensitivity analysis restricting statin users to those with statin use at ADT initiation further reinforced the analysis's validity and minimized any effects that the timing of statin use may have had on the observations.

Although there is laboratory evidence suggesting that statins may delay the development of castration resistance,^28^ clinical evidence is far from conclusive. In a post hoc analysis of RCT data, Hamilton et al.^36^ did not find any notable differences in the timeframe for developing CRPC among patients experiencing biochemical recurrence after radiotherapy. Conversely, a retrospective study conducted by Jung et al.^37^ provided support for the potential use of statins in delaying the progression to CRPC in patients with metastatic PCa. These highlight the need for further studies in this area. Furthermore, few studies have compared the effects on mortality between hydrophilic and lipophilic statins. While initial reports suggested that hydrophilic statins may possess stronger protective effects,^38^ most existing studies only included patients receiving lipophilic statins,^39^, ^40^ probably due to the relatively high potency of lipophilic statins and their consequently limited indications. For the same reason, while the present study included a mix of hydrophilic and lipophilic statins, the number of patients on hydrophilic statins was too small for any meaningful subgroup analysis to be performed. This knowledge gap is still unresolved and awaits additional investigations.

### 4.3 Strengths and limitations

This study employed an extensive and representative database covering a wide geographic area, with follow‐up for a substantial duration. As a result, the findings are broadly applicable and mirror real‐world medical practices. Furthermore, we conducted sensitivity analyses using various methods, which consistently yielded similar results, indicating the reliability and strength of our findings. However, it is important to acknowledge several limitations of this study. Being an observational study, the possibility of residual confounding cannot be ruled out. Moreover, the diagnostic data included in the study could not be independently verified. Nevertheless, it is worth noting that the codes used were entered by treating clinicians for clinical purposes, independent of the authors. Although this observational study has a considerable duration of follow‐up, longer‐term associations remain unknown. In addition, the results may not be generalizable to non‐Asian populations. These necessitate further studies. Furthermore, a small subset of patients (*N* = 102) lacked an initial PSA measurement. Nonetheless, sensitivity analysis adjusting for baseline PSA measurement where available demonstrated that the results were robust. As the number of patients with missing baseline PSA measurements was relatively small, this sensitivity analysis should sufficiently demonstrate the robustness of the reported results. Moreover, our findings may be affected by the “healthy user effect” as well, as it is possible that patients who were adherent to statin therapy may also be more adherent to PCa treatment, and therefore, further studies, especially RCTs, are needed to identify if statins can improve mortality risk in PCa patients. Finally, due to limitations inherent in the database, specific information regarding cancer staging and grading was not available. To compensate for this limitation, we employed the use of androgen receptor antagonists or chemotherapy as a proxy for metastatic PCa. However, it is essential to conduct future studies with more detailed data to further explore this aspect. Finally, future work should consider building personalized predictive models specifically for PCa patients for better risk stratification, as performed for other diseases.^41^

## 5 CONCLUSIONS

The concurrent use of statins and ADT in Asian patients with PCa was found to be significantly associated with reduced risks of both PCa‐related mortality and all‐cause mortality. These associations may be particularly prominent in patients with less advanced PCa.

## AUTHOR CONTRIBUTIONS

**Yan Hiu Athena Lee:** Conceptualization (lead); data curation (lead); methodology (lead); validation (lead); writing – original draft (lead); writing – review and editing (lead). **Jeffrey Shi Kai Chan:** Conceptualization (lead); methodology (lead); validation (lead); writing – original draft (lead); writing – review and editing (lead). **Jeremy Man Ho Hui:** Conceptualization (lead); formal analysis (lead); methodology (lead); software (lead); validation (lead); visualization (lead); writing – original draft (lead); writing – review and editing (lead). **Pias Tang:** Writing – review and editing (equal). **Kang Liu:** Data curation (equal); writing – review and editing (equal). **Edward Christopher Dee:** Writing – review and editing (equal). **Kenrick Ng:** Writing – review and editing (equal). **Gary Tse:** Project administration (equal); supervision (equal); writing – review and editing (equal). **Chi Fai Ng:** Project administration (equal); supervision (equal); writing – review and editing (equal).

## FUNDING INFORMATION

None.

## CONFLICT OF INTEREST STATEMENT

ECD is funded in part through the Cancer Center Support Grant from the National Cancer Institute (P30 CA008748). GT is supported by a Research Impact Fund from Hong Kong Metropolitan University (Project Reference No. RIF/2022/2.2). All other authors declare no disclosure of interest for this contribution.

## ETHICS STATEMENT

This study has been approved by the Joint Chinese University of Hong Kong—New Territories East Cluster Clinical Research Ethics Committee.

## Supporting information


**Tables S1–S6**.

## Data Availability

Data will be made available upon reasonable requests.
