# Supplementary material for: Statin use and mortality risk in Asian patients with prostate cancer receiving androgen deprivation therapy: A population‐based cohort study
Source: Cancer Med. 2023 Dec 22;13(1):e6826. doi: 10.1002/cam4.6826 (PMC10807587; doi:10.1002/cam4.6826)
Supplement: Supplementary file 1 — Tables S1–S6. [file CAM4-13-e6826-s001.docx]

**Supplementary Table 1.** International Classification of Diseases, Ninth Revision **(**ICD-9) codes used to identify outcomes and co-morbidities. All hereby listed codes include the corresponding sub-codes.

| Prostate cancer | 185 |
| --- | --- |
| Diabetes mellitus | 250 |
| Anemia | 280-283, 284.0, 284.1, 284.8, 284.9, 285 |
| Heart failure | 428 |
| Myocardial infarction | 410 |
| Hypertension | 401 402 403 404 405 437.2 |
| Atrial fibrillation | 427.31 |
| Stroke or transient ischaemic attack | 430 431 432 433 434 435 |
| Chronic obstructive pulmonary disease | 490 491 492 496.0 |
| Ischaemic heart disease | 410 411 412 413 414 |
| Chronic kidney disease | 582 585 586 |
| Chronic liver disease | 571 |
| Hyperlipidaemia | 272.0 272.1 272.2 272.3 272.4 |
| Malignancy | 140 141 142 143 144 145 146 147 148 149 150 151 152 153 154 155 156 157 158 159 160 161 162 163 164 165 170 171 172 173 174 175 179 179 180 181 182 183 184 185 186 187 188 189 190 191 192 193 194 195 196 197 198 199 200 201 202 203 204 205 206 207 208 209.0 209.1 209.2 209.3 |

**Supplementary Table 2.** International Classification of Diseases, Ninth Revision **(**ICD-9) and International Classification of Diseases, Tenth Revision (ICD-10) codes used to identify outcomes and co-morbidities. All hereby listed codes include the corresponding sub-codes.

| Prostate cancer-related mortality | ICD-9: 185  ICD-10: C61 |
| --- | --- |

**Supplementary Table 3.** Weighted comparisons of outcomes by statin use with subgroups for the type of androgen deprivation therapy. Weighted hazard ratios (wHR) with corresponding 95% confidence intervals (CI) were calculated, using statin non-users as the reference group.

|  | BO only (N=1681) | | GnRH agonists or antagonists only (N=2870) | | Both BO and GnRH agonists or antagonists (N=369) | |
| --- | --- | --- | --- | --- | --- | --- |
|  | wHR [95% CI] | p value | wHR [95% CI] | p value | wHR [95% CI] | p value |
| Prostate cancer-related mortality | 0.47 [0.38, 0.59] | <0.001 | 0.60 [0.48, 0.76] | <0.001 | 0.70 [0.41, 1.19] | 0.188 |
| All-cause mortality | 0.48 [0.42, 0.55] | <0.001 | 0.63 [0.55, 0.73] | <0.001 | 0.62 [0.42, 0.91] | 0.015 |

BO, bilateral orchidectomy. GnRH, gonadotropin-releasing hormone.

**Supplementary Table 4.** Sensitivity analysis including only patients who had statin exposure at the time of androgen deprivation therapy initiation as the user group (total N=4436). Weighted comparisons of outcomes by statin use were presented. Weighted hazard ratios (wHR) with corresponding 95% confidence intervals (CI) were calculated, using statin non-users as the reference group.

|  | wHR [95% CI] | p value |
| --- | --- | --- |
| Prostate cancer-related mortality | 0.64 [0.54, 0.75] | <0.001 |
| All-cause mortality | 0.68 [0.61, 0.76] | <0.001 |

**Supplementary Table 5.** Sensitivity analysis excluding patients who had any hydrophilic statin exposure from the user group (total N=4686). Weighted comparisons of outcomes by statin use were presented. Weighted hazard ratios (wHR) with corresponding 95% confidence intervals (CI) were calculated, using statin non-users as the reference group.

|  | wHR [95% CI] | p value |
| --- | --- | --- |
| Prostate cancer-related mortality | 0.57 [0.49, 0.67] | <0.001 |
| All-cause mortality | 0.58 [0.52, 0.64] | <0.001 |

**Supplementary Table 6.** Sensitivity analysis including only patients with three years or more of follow up (total N=2661). Weighted comparisons of outcomes by statin use were presented. Weighted hazard ratios (wHR) with corresponding 95% confidence intervals (CI) were calculated, using statin non-users as the reference group.

|  | wHR [95% CI] | p value |
| --- | --- | --- |
| Prostate cancer-related mortality | 0.68 [0.52, 0.88] | 0.003 |
| All-cause mortality | 0.67 [0.58, 0.76] | <0.001 |
